# Comparison of three activity monitors for estimating sedentary time among children

**DOI:** 10.1186/s13102-016-0028-y

**Published:** 2016-02-05

**Authors:** Jarle Stålesen, Frøydis Nordgård Vik, Bjørge Herman Hansen, Sveinung Berntsen

**Affiliations:** Department of Public Health, Sport and Nutrition, Faculty of Health and Sport Sciences, University of Agder, P.O. Box 422, NO-4604 Kristiansand, Norway; Department of Sports Medicine, Norwegian School of Sport Sciences, Oslo, Norway

**Keywords:** Accelerometer, ActiGraph, ActivPAL, Inclinometer, Indirect calorimetry, SenseWear

## Abstract

**Background:**

Time spent sedentary appears to be associated with several health outcomes in adults, but findings are inconsistent in children. Further, the assessment of sedentary time represents a major challenge. The objectives of the present study were to determine whether 1) ActiGraph GT3X+, ActivPAL and SenseWear Armband Pro3 (SWA) provide comparable estimates of sedentary time in 9–12-year-old children, 2) these devices are valid compared with direct observation, and 3) ActivPAL discriminates between sitting and standing behavior.

**Methods:**

The sample was 67 children. Data were collected during three consecutive days in November 2012. To test the activity monitors in contexts related to physical and sedentary activities commonly performed by children, the children participated in sessions of activity while sitting (watching television, playing video games and tossing a ball while sitting) and standing (musical chairs, active video gaming and tossing a ball) while wearing three different activity monitors at the same time. All activity sessions were observed by two researchers. Differences between monitors were determined using Friedman’s two-way analysis of variance by rank order.

**Results:**

Minutes of estimated sedentary time differed across device brands during combined sitting activities: SWA vs. ActiGraph GT3X+ (*P* = 0.048), SWA vs. ActivPAL, (*P* < 0.001) and ActiGraph GT3X+ vs. ActivPAL (*P* = 0.002). Out of 12 min in total of combined recorded sitting activity, SWA reported a median of 6 min (95 % Confidence Interval [CI] = 5.0, 7.0), ActiGraph GT3X+ 7 min (7.0, 8.0) and ActivPAL 10 min (8.6, 10.8) as sedentary time. ActivPAL recorded 3.7 (2.4, 4.0) minutes of the non-sitting activities ‘musical chairs’, 4.0 (4.0, 4.0) minutes in ‘standing ball toss’; and 4.0 (2.7, 4.0) minutes in ‘active video gaming’ as sitting time.

**Conclusion:**

Recorded sedentary time varied among the monitors GT3X+, SWA and ActivPAL, and misclassification of standing activities as sitting activities were apparent for ActivPAL in certain activities.

## Background

The word “sedentary” comes from the Latin *sedentarius*, meaning sitting or remaining in one place. Sedentary time is associated with detrimental health outcomes in adults [1], but whether this is the case in children is uncertain [2]. Sedentary time has been inversely associated with high-density lipoprotein cholesterol in overweight and obese children [3] and self-reported screen time has been associated with pediatric obesity and cardio metabolic disease [4].

Previously, screen time has often been used to quantify sedentary time [5–7]. However, this may be inadequate, given that sedentary time includes more than screen-based activities [8]. In other studies in which accelerometers are used, associations are not always found between objectively recorded sedentary time and health risks [8–10], especially when adjusting for moderate- to vigorous intensity physical activity [10]. Thus, the relation between sedentary time and health risk may not be as clear as it is between physical activity and health. Findings are also complicated by researchers’ use of different definitions of sedentary [8]. Further understanding the objective relations between sedentary time and health risks in children is therefore dependent on the use of valid assessment methods. Accelerometers and inclinometers are now commonly used to objectively record total sedentary time [8, 11, 12]. ActiGraph GT3X+ (ActiGraph, Pensacola, FL, USA), a hip-worn accelerometer, ActivPAL™ (ActivPAL Technologies Ltd., Glasgow, UK), an inclinometer worn on the leg between the knee and hip, and SenseWear Armband Pro3 (SWA, BodyMedia Inc., Pittsburgh, PA, USA), a multisensor activity monitor worn on the arm, are three commonly used activity monitors that have been used previously to record sedentary time in children [13–15].

To our knowledge there are no published comparisons of GT3X+, ActivPAL and SWA for recording sedentary time in children. In a study comparing reports of sedentary time with ActivPAL and GT3X+ over a seven-day period in preschool children, a correlation between these monitors of *r* = 0.66 (*P* = 0.001) was reported [16]. GT3X+ and ActivPAL have also been compared with direct observation in 15–18-year-old females [17]. The overall agreements with direct observation were 67 % for GT3X+ and 99 % for ActivPAL across sitting, standing and slow walking (3.6 km/h) [17].

In several studies comparing older generations of SWA with indirect calorimetry, SWA underestimated energy expenditure during sedentary time in children [12, 18]. Calabro et al. [19] compared SWA with indirect calorimetry in 21 children during various activities including coloring and computer games and found no significant differences between SWA and indirect calorimetry [19].

When evaluating the validity and reliability of activity monitors, it is essential to assess relevant activities in a free-living setting. Although few studies have evaluated the validity of activity monitors during activities out of a laboratory [13] or during both out and in a laboratory [20], the majority have been laboratory based [21–23].

The objective of the present study were to 1) determine whether the GT3X+, ActivPAL and SWA provided comparable estimates of sedentary time in 9–12-year-old children, 2) evaluate the validity of these device estimates against direct observation and 3) investigate whether ActivPAL could discriminate between sitting and standing activities.

## Methods

### Design

Data were collected during three consecutive days in November 2012. To test the activity monitors in contexts related to physical and sedentary activities commonly performed by children, 67 children participated in sitting and standing activities in random order while simultaneously wearing all three activity monitors. Two researchers observed each activity. Written informed consent was obtained from the participating children and their parents or guardians. Application was sent to the Norwegian regional committee for medical and health research ethics South East (2012/1803) and the committee advised running the study without further approval. The specificity of activities the children participated in was carefully arranged, and the purpose of the observers was to write down any deviation to the pre-determined activity on a time sheet.

### Subjects

In the present study, 35 boys and 32 girls were recruited from an elementary school in Kristiansand, Norway. The total number of children asked to participate was 27 in 5^th^ grade, 26 in 6^th^ grade and 24 7^th^ grade, respectively. No children declined to participate, and the reason for those that did not participate was absence from school during the days of testing. Eligible participants were between the ages of 9 and 12 years. Due to technical errors when downloading data, 13 children were excluded (five with GT3X+, four with ActivPAL and four with SWA). This resulted in a total sample of 54 children (27 boys and 27 girls).

### Procedures

The researchers followed the participants in groups of two and recorded their age, gender, body mass and time intervals for each activity on a standardized form. The test location was a classroom. Each child’s body mass was measured to the nearest 0.1 kg using a Seca Optima (Seca, Hamburg, Germany). Prior to testing, observers’ watches were synchronized with the computer clock. The total duration of collection of data per child was approximately 1 hour. Physical activity data was collected in a consecutive way while participating in the following activity stations.

The ActiGraph GT3X+ is an activity monitor with a triaxial accelerometer that has previously been described in detail [24]. Data were collected at a sampling rate of 100 Hz, and analyzed in 60-s epochs. When analyzing the data, we derived counts per minute and minutes of intensity-specific physical activity from the vertical axis data using the youth-specific cutoffs proposed by Evenson et al. [21] of less than 100 counts per minute defined as sedentary time. ActiLife v.6.8.0 (Pensacola, FL, USA) software was used for analyses.

The ActivPAL™ physical activity logger is an inclinometer that can discriminate between and record time spent in different postures over a 7-day period.

The ActivPAL has previously been described in detail [25, 26]. Date were collected in 15-s epochs with a sampling frequency of 10 Hz and analyzed in 60-s epochs. When comparing ActivPAL with GT3X+ and SWA, time registered as sitting was categorized as sedentary time, while standing and stepping time was categorized as other activities. ActivPAL™ data were processed and analyzed using Research Edition v6.5.1 (Glasgow, UK).

The SenseWear Armband Pro_3_ (SWA) is a multisensor activity monitor worn over the triceps of the right arm. The SWA has been described in detail previously [13, 27]. The sampling frequency was 1-min epochs. Time spent sedentary was defined as metabolic equivalents (METs) below 1.5 and time spent in other activities as 1.5 METS or above for the SWA. The SenseWear Professional 6.1 software was used to analyze raw data.

ActivPAL, SWA and GT3X+ were initialized and attached to the child according to the manufacturers’ instructions.

To quantify time spent in a wide range of physical activities, including light intensity and sedentary time, six activities were used: standing ball toss, sitting ball toss, musical chairs, television viewing, sedentary video gaming and active video gaming. The individual activities are described more in detail in Table 1. Activities were further divided into those performed sitting or standing. Standing activities included ball toss, musical chairs and active video gaming. Television viewing, sitting ball toss and sedentary gaming were sitting activities. Each activity lasted 6 min and was performed in a randomized order.

**Table 1:** Summary of the six activity stations

Station 1: Standing Ball- toss	Two children stood upright in the same spot for the duration of the test and threw a rubber ball back and forth over a distance of approximately 2.5 m.
Station 2: Sitting Ball- toss	Two children performed this exercise in a sitting position facing each other, and threw a rubber ball over a distance of approximately 2 m.
Station 3: Musical chairs	The test group prepared a round table, 1.5 m in diameter. Then added one chair, made the children walk in circles around the table, at a low pace with a fixed distance between. The children sat down and stood up again when the test leader notified. The children were sitting no more than 10 s in each interval.
Station 4: Television viewing	The children watched television sitting.
Station 5: Sedentary gaming	The children were sitting in front of a television and played a video game using a handheld controller.
Station 6: Active Video gaming	The children were standing upright in front of a television, playing a video game with a motion controller.

### Statistical analysis

Descriptive data are presented as mean and standard deviation (SD). Results are presented as medians and 95 % confidence intervals (CIs). Due to skewness, nonparametric tests (related-samples Friedman’s two-way analysis of variance by rank order) were performed. The first and last minutes of each session were excluded, leaving the middle 4 minutes of each session for analyses. The criterion for statistical significance was *P* ≤ 0.05. Analyses were conducted using SPSS® (Statistical Package for Social Sciences, Version 22 for Windows. SPSS Inc., Chicago, USA).

## Results

A total of 54 children (27 boys, 11.1 ± 0.7 years) with a mean body mass of 41.9 ± 9.6 kg were included. Median sedentary times recorded by each of the three monitors during activities are summarized in Table 2. ActivPAL recorded significantly more sedentary time (*P* < 0.001) during four of the six activities compared with both SWA and GT3X+. Out of the possible 4 min, ActivPAL recorded 3.7 (95 % CI 2.4, 4.0) minutes as sedentary time during musical chairs, 4.0 (2.7, 4.0) minutes during active video gaming and during standing ball toss 4.0 (4.0, 4.0). As shown in Table 2, SWA and GT3X+ were significantly different (*P* = 0.018) from each other for sitting ball toss, during which SWA recorded 0.0 min (0.0, 1.0) and GT3X+ recorded 0.5 min (0.0, 1.0) as sedentary time. ActivPAL was also significantly different from each of the other monitors (*P* < 0.001), recording 4.0 min (3.3, 4.0) as sedentary time in sitting ball toss.

**Table 2:** Median (95 % confidence intervals) of minutes spent sedentary for SWA, GT3X+ and ActivPAL (APAL) (*n* = 54)

	Standing ball- toss	Sitting ball- toss	Musical chairs	Television viewing	Sedentary gaming	Active video gaming
SWA	0.0 (0.0, 0.0)	0.0 (0.0, 0.0)*	0.0 (0.0, 0.0)	4.0 (2.0, 4.0)	4.0 (3.0, 4.0)	0.0 (0.0, 0.0)
GT3X+	0.0 (0.0, 0.0)	0.5 (0.0, 1.0)*	0.0 (0.0, 0.0)	3.0 (2.0, 4.0)	4.0 (4.0, 4.0)	0.0 (0.0, 0.0)
APAL	4.0 (4.0, 4.0)**	4.0 (3.3, 4.0)**	3.7 (2.4, 4.0)	4.0 (3.2, 4.0)	4.0 (4.0, 4.0)**	4.0 (2.7, 4.0)**

*Significantly different compared to the other monitors (*P* < 0.05)

**Significantly different compared to the other monitors (*P* < 0.001)

The activities lasted 4 min in total

**Fig. 1:**
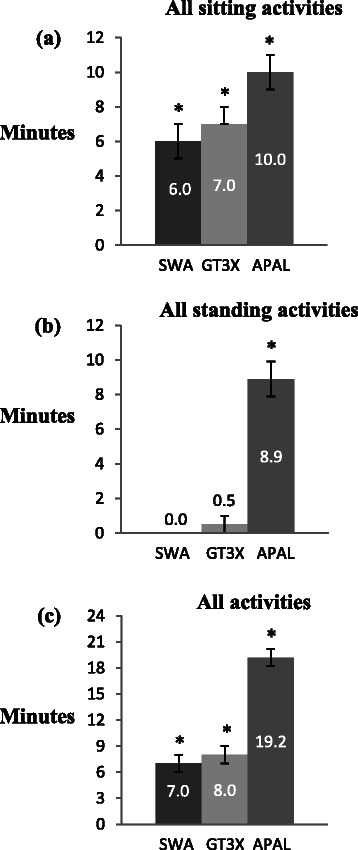
Presenting the median sedentary minutes (95 % CI) for SWA, GT3X+ and ActivPAL. Detailed legend: All sitting- (**a**) and standing (**b**) activities were compared, with a total of 12 min. All activities (**c**) were compared with a total of 24 min. In these figures *n* = 54

When stratified into standing activities (Fig. 1), ActivPAL recorded significantly different sedentary time (*P* < 0.001) to that recorded by SWA or GT3X+. In the standing activities ActivPAL recorded that the children were sedentary with a median of 8.9 (8.4, 9.8) out of 12 min in total. During the sitting activities (Fig. 1), there were significant differences between all three monitors (SWA vs. GT3X+, *P* = 0.048; SWA vs. ActivPAL, *P* < 0.001; GT3X+ vs. ActivPAL, *P* = 0.002). ActivPAL recorded 10 min (8.6, 10.8) of sedentary time while SWA recorded 6 min (5.0, 7.0) and GT3X+ 7 min (7.0, 8.0) out of a possible 12 min during the sitting activities (Fig. 1).

Table 3 presents the amount of time recorded by ActivPAL as sitting, standing and stepping in all activity stations. For standing ball toss, musical chairs, and active video gaming, ActivPAL recorded 4 min (4.0, 4.0), 3.7 min (2.4, 4.0) and 4 min (2.7, 4.0) respectively, out of 4 min in total as sitting

**Table 3:** Median (95 % confidence intervals) of minutes spent sitting, standing and stepping for AcitvPAL (*n* = 54)

	Standing ball- toss	Sitting ball- toss	Musical chairs	Television viewing	Sedentary gaming	Active video gaming
Sitting	4.0 (4.0, 4.0)	4.0 (3.3, 4.0)	3.7 (2.4, 4.0)	4.0 (3.2, 4.0)	4.0 (4.0, 4.0)	4.0 (2.7, 4.0)
Standing	0.0 (0.0, 0.0)	0.0 (0.0, 0.6)	0.3 (0.0, 1.4)	0.0 (0.0, 0.6)	0.0 (0.0, 0.0)	0.0 (0.0, 0.9)
Stepping	0.0 (0.0, 0.0)	0.0 (0.0, 0.1)	0.0 (0.0, 0.2)	0.0 (0.0, 0.1)	0.0 (0.0, 0.0)	0.0 (0.0, 0.2)

The activities lasted 4 min in total

## Discussion

We found that activity monitors differed in their recordings of sedentary time. All three activity monitors recorded sedentary time significantly different during sitting activities. ActivPAL recorded sedentary time more accurately compared with SWA and GT3X+. However, in our study ActivPAL seemed to have difficulty discriminating between sitting activities and standing or moving exercises, and therefore misclassified standing or moving exercises as time spent sitting.

Our findings differ somewhat from previous reports indicating that ActivPAL is valid for discriminating between sedentary, standing and walking activities [17, 22, 25]. Davis et al. [25] reported that ActivPAL misclassified posture in a few individuals, most often recording sitting as standing activities, although they did not state the number of participants to which this applied. It was reported that occasionally standing was misidentified by ActivPAL as sitting, for example if a child stood with one leg straight and one leg bent at the knee with the foot resting on top of the other foot, thereby altering the angle [25]. These findings resembles ours; however, it is unlikely any child in the present study rested one foot on top of the other in the standing activity where we tested the inclinometer, as that most likely would compromise the balance required to throw a ball in standing ball toss, confirmed by direct observation. The angle of the leg; however, may explain some of the misclassifications by the ActivPAL. When comparing ActivPAL’s ability to record sitting time compared with direct observation, it accurately recorded sitting ball toss, television viewing and sedentary gaming as sedentary activities.

Similar to ActivPAL’s reported occasional misidentification when a child stood with one leg straight and one leg resting on top of the other [25]; SWA recorded 0 min as sedentary time, in sitting ball toss activity, a station involving marked arm movement. This may be explained by the location of activity monitor on the arm and the arm movement. Similar explanation was reported in a previous study as a possible cause of overestimation of energy expenditure by SWA [28].

It has been argued that using various ActiGraph cut-offs provides distinct estimates of sedentary time [16]. This is a challenge when comparing our results with those of previous studies of GT3X+. For instance, Martin et al. [16] defined sedentary cut-offs as <1100 counts per minute, while in the present study we used <100 counts per minute as recommended in studies comparing various cut-offs for GT3X+ [29, 30] . In a study examining the validity of seven child-specific ActiGraph cut-offs using indirect calorimetry as the criterion, the majority overestimated sedentary time and underestimated moderate to vigorous physical activity in children [20].

In two studies comparing GT3X+ and ActivPAL, ActivPAL was more valid for both recording sedentary time [16, 17] and distinguishing between sitting and standing [17]. The results of the present study support the accuracy of ActivPAL in recording sitting time but show that it may overestimate sedentary time and partly misclassify standing activities as sitting.

Inclinometers and accelerometers may be promising tools in lifestyle intervention studies. However, due to the observed differences in how monitors record sedentary and sitting time, we recommend additional research. A more comprehensive exploration of the strengths and weaknesses of different activity monitors in both laboratory and natural settings may provide a better understanding of the relation between sedentary time and health.

Study strengths were the inclusion of multiple monitors to record sedentary time and the fact that activities were conducted in a natural rather than a laboratory-based setting. Our study included activities such as watching television and sedentary gaming, both of which are common leisure-time activities that contribute significantly to children’s total sedentary time. The activities were also presented in a randomized order. Furthermore, the use of direct observation to verify the children’s adherence to the study protocol and the ways in which they performed the activity strengthened the study.

A primary study limitation was definition of sedentary time for ActivPAL versus SWA and GT3X+. When comparing ActivPAL with SWA and GT3X+, we did not include standing without movement as part of sedentary time. However, consideration should be given to the different definitions of sedentary time upon which the three monitors are based, i.e. ActivPAL records sitting, standing without movement and walking or stepping, while GT3X+ and SWA primarily record movements of various body parts. Standing without movement in the definition of sedentary time for ActivPAL could have been included based on that SWA and GT3X+ were designed to record movement, as in previous research comparing ActivPAL with GT3X+ [16]. However, ActivPAL was designed to record posture, and including standing without movement as sedentary time would have been to take away ActivPAL’s purpose. To further investigate whether ActivPAL discriminates between sitting, standing and stepping activities, ActivPAL was individually compared with direct observation.

## Conclusion

In conclusion, children’s sedentary time recorded simultaneously by GT3X+, SWA and ActivPAL differed significantly. Recorded sedentary time varied among the monitors GT3X+, SWA and ActivPAL and misclassification of standing activities as sitting activities were apparent for ActivPAL in certain activities.
